# Off the Line

**Published:** 1909-12-04

**Authors:** Walter K. Alvey


*To the Editor of* The Hospital.

### "OFF THE LINE."

*To the Editor of* The Hospital.

Sir,—I have been reading the comments in your last number on my speech at the annual dinner of the Hospital Officers' Association, and it appears that the member of your editorial staff responsible for those comments is very angry with me. One sentence of mine has so roused his indignation that he devotes two entire columns to reproving me, and finally, having, I presume, exhausted his own powers, he hands me over to the vengeance of offended Deity.

He is angry, in the first instance, on behalf of the Hospital Officers' Association, the members of which will doubtless be profoundly moved by this touching manifestation of his solicitude. Now the passage which he quotes —or rather misquotes—from the November issue of the *Hospital Gazette* is simply, as he could easily see, an expression of the writer's personal opinion, and everything in my speech was simply an expression, more or less crude and inadequate, of my personal opinions. I transgressed by my remarks no rule or principle of conduct of the Association, written or unwritten, nor did I—and indeed I could not—in any way pledge the Association to the views to which I gave utterance.

Secondly, the writer of the comments is angry because I said something with which *he* does not agree, and which is "directly opposed to the opinion of those who are best entitled to speak on behalf of the voluntary hospitals of this country." Then why this rage? If he is so convinced that he is right, and that he has on his side all those who are best entitled to speak on the question, he can surely afford to smile indulgently on my position of harmless isolation.

Next, I am told that my views ill become the Secretary of Charing Cross Hospital. Of course I was speaking not as Secretary of Charing Cross Hospital, but merely as a member of the Association to members of the Association; yet, even so, I said nothing of which the Secretary of Charing Cross Hospital should feel ashamed. The fact is—and I say this for the benefit of those of your readers who may have been misled—I am not the wild iconoclast depicted in your columns. I did not say, or even hint, that I wished for the destruction of the voluntary system. What I did say—but no, if your readers are really anxious to know that, they will find, I believe, an accurate report in the next issue of the *Hospital Gazette*. The writer of the comments surely knows already, for, although he has confined his attention to one brief remark—the only one, so far as I know, publicly reported—I do not like to think that he has ventured to criticise my speech after reading that remark alone. Yet at the same time I find it hard to understand how, with the speech before him, he could, except with deliberate intention, so utterly misrepresent me. Both these alternatives are so repugnant to one's sense of honour and fair play that I suppose there must be some better explanation, which I patiently await.

Yours faithfully,

Walter K. Alvey.

[We cannot permit Mr. Alvey to ride off from the position he took up, as reported in the press, by attributing anger to the writer of our notes last week. We publish his letter as illustrating a spirit which, if allowed to dominate the controllers of the Hospital Officers' Association, will render it impossible for the more prudent and statesmanlike secretaries and officers of hospitals to continue to belong to it. No one with any sense of responsibility meets an argument supported by facts in the petty personal spirit exhibited by Mr. Alvey in his letter. He does not attempt to meet a single one of the points we raised, nor does he deny the accuracy of the quotation from his speech, as reported, which we republished. Petty personal points like those Mr. Alvey raises are unworthy of a discussion upon a great question which involves the maintenance or destruction of the voluntary principle of hospital support. We may point out, however, that the *Hospital Gazette* is declared on its title page to be the official journal of the Hospital Officers' Association, and that its wise counsel, which Mr. Alvey has ignored, was contained in a leading article, and is not, as he states, merely an expression of one writer's personal opinion. There must be discipline in an association of this kind, and a vice-president is the first person who ought to enforce it by his example. Mr. Alvey cannot, nor can any member of the Association, who speaks in public on hospital questions, divest himself, as Mr. Alvey attempts to do, of the official office which he holds. It is for this reason that the wiser heads in the Hospital Officers' Association feel, as the official *Gazette* properly insists, that its members should abstain from public discussion of the high politics of the hospital world. Mr. Alvey's attitude in the present discussion will strengthen this view, and make its wisdom apparent to everybody.—Ed. The Hospital.]

